# Biodegradable and Biocompatible Polyhydroxy-alkanoates (PHA): Auspicious Microbial Macromolecules for Pharmaceutical and Therapeutic Applications

**DOI:** 10.3390/molecules23020362

**Published:** 2018-02-08

**Authors:** Martin Koller

**Affiliations:** 1 Office of Research Management and Service, c/o Institute of Chemistry, University of Graz, NAWI Graz, Heinrichstrasse 28/III, 8010 Graz, Austria; martin.koller@uni-graz.at; Tel.: +43-316-380-5463; 2 Association for Resource Efficient and Sustainable Technologies—ARENA, Inffeldgasse 21b, 8010 Graz, Austria

**Keywords:** biocompatibility, biodegradability, biopolyesters, biopolymers, composites, drug release, implants, polyhydroxyalkanoates, scaffolds, tissue engineering

## Abstract

Polyhydroxyalkanoates (PHA) are bio-based microbial biopolyesters; their stiffness, elasticity, crystallinity and degradability are tunable by the monomeric composition, selection of microbial production strain, substrates, process parameters during production, and post-synthetic processing; they display biological alternatives for diverse technomers of petrochemical origin. This, together with the fact that their monomeric and oligomeric *in vivo* degradation products do not exert any toxic or elsewhere negative effect to living cells or tissue of humans or animals, makes them highly stimulating for various applications in the medical field. This article provides an overview of PHA application in the therapeutic, surgical and tissue engineering area, and reviews strategies to produce PHA at purity levels high enough to be used *in vivo*. Tested applications of differently composed PHA and advanced follow-up products as carrier materials for controlled *in vivo* release of anti-cancer drugs or antibiotics, as scaffolds for tissue engineering, as guidance conduits for nerve repair or as enhanced sutures, implants or meshes are discussed from both a biotechnological and a material-scientific perspective. The article also describes the use of traditional processing techniques for production of PHA-based medical devices, such as melt-spinning, melt extrusion, or solvent evaporation, and emerging processing techniques like 3D-printing, computer-aided wet-spinning, laser perforation, and electrospinning.

## 1. Introduction

Polyhydroxyalkanoates (PHA) are prokaryotic storage macromolecules; they are accumulated as water insoluble granules in the cytoplasm of numerous bacteria and several extremophilic archaea. PHA granules are also proposed to be referred to as “carbonosomes” by some leading scientists in this field in order to underline their complex biological functions [1]. The presence of PHA in microbial cells assists survivability of microbes under famine and environmentally challenging conditions [2,3]. Chemically, PHA are *in vivo* polymerized polyoxoesters of hydroxyalkanoates, with the hydroxyl group being typically located at the monomers’ β-carbon atom. Biodegradability is not the only beneficial feature of PHA; moreover, they display high biocompatibility. This is well visible by the natural occurrence of PHA building blocks like 3-hydroxybutyrate (3HB) and related oligomers in the blood stream of humans and animals (reviewed by [4,5,6,7]). In addition, monomers and oligomers of hydroxyalkanotes derived from natural aliphatic PHA and synthetic analogues are reported to exert bioactive functions [8]. In the context of biobased polyester oligomers, Utsunomia and colleagues recently reported on the production of oligomers consisting of lactate and 3HB; these oligomers were produced and excreted using recombinant *Escherichia coli*; co-feeding of *E. coli* with diethylene glycol resulted in the formation of lactate-3HB oligomers with hydroxyl termini. These products can undergo polyaddition reaction with diisocyanate, yielding lactate-3HB-based poly(ester-urethane) as a novel group of biobased polyesters with supposed intriguing properties [9].

**Figure 1:**
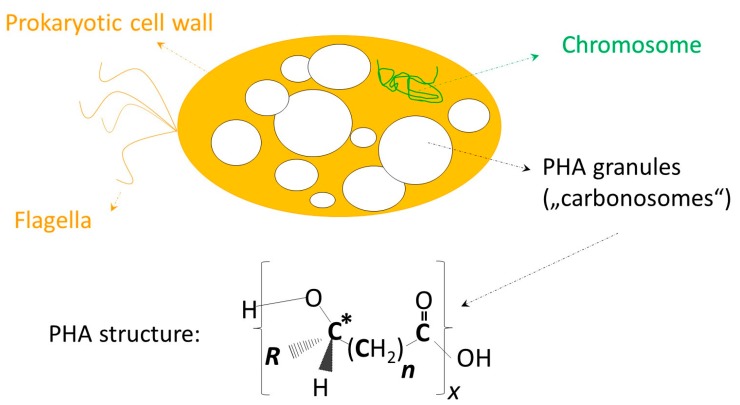
General chemical structure of polyhydroxyalkanoates (PHA). The upper part of the illustration symbolizes a microbial cell containing PHA as granular inclusion bodies (“carbonosomes”). ***R*** displays the side chain of PHA monomers, ***n*** the number of methylene groups in the monomers’ backbones, and ***x*** represents the degree of polymerization. The asterisk (*) indicates the chiral center of most monomers. PHA building blocks (monomers) discussed in this review: *scl*-PHA building blocks: *R* = CH_3_, *n* = 1: 3-hydroxybutyrate (3HB); *R* = H, *n* = 2: 4-hydroxybutyrate (4HB) (achiral!); *R* = C_2_H_5_, *n* = 1: 3-hydroxyvalerate (3HV); *mcl*-PHA building blocks: *R* = C_3_H_7_, *n* = 1: 3-hydroxyhexanoate (3HHx); *R* = C_4_H_9_, *n* = 1: 3-hydroxyoctanoate (3HO); *R* = C_4_H_8_, *n* = 1: 3-hydroxy-ω-heptenoate (unsaturated); *R* = C_8_H_16_, *n* = 1: 3-hydroxy-ω-undecenoate (unsaturated).

From a technological perspective, PHA attract attention due to their thermoplasticity, their production starting from abundantly available renewable resources, their biodegradability, and their biocompatibility. Currently, the integration of PHA production processes into biorefinery concepts and waste treatment facilities is heavily examined in order to make these processes efficient both in sustainability and economic terms [10,11,12]. Composition on the level of monomeric building blocks, microstructure, and supra-macromolecular architecture determine the chemo-mechanical properties of PHA, and thus their suitability for defined technological applications [13]. Dependent on the monomeric composition, we differentiate short-chain-length PHA (*scl*-PHA) consisting of monomers with three to five carbon atoms (*nota bene*: two to five when including also glycolate as PHA-related building block), and medium-chain-length PHA (*mcl*-PHA) with six and more carbon atoms per monomer. In this context, rather crystalline *scl*-PHA feature typical thermoplastic properties, whereas *mcl-*PHA resins resemble elastomers and latex-like materials [4]. Based on the biotechnological production strategy, PHA constitute either homopolyesters, which consist of only one type of monomer, or heteropolyesters. Heteropolyesters in turn are grouped into copolyesters (monomers of either different backbones or side chains, *cf.* Figure 1) and terpolyesters (consisting of monomers differing in both their side chains and backbones) [4,5,6]. Production of PHA heteropolyesters typically requires supplying of substrates structurally related to the desired building blocks. For example, incorporation of 3-hydroxyvalerate (3HV) monomers into the rather crystalline matrix of poly(3-hydroxybutyrate) (PHB) homopolyester was reported in cultivations supplied with molecules structurally related to 3HV, such as propionate [14] or valerate [15], levulinic acid [16,17], or ozonolysis products of fatty acids [18]; to synthesize 4-hydroxybutyrate (4HB), the only important achiral PHA constituent, the supply of 4HB-related precursors like γ-butyrolactone was reported [19,20]. Exceptions are found among production strains like *Haloferax mediterranei*, which produce 3HV from unrelated sources like sugar or glycerol [21].

In the field of medicine, a major arena for application of different polymers, well-established polymeric products usually are not the materials of choice to meet the requirements of material performance, biocompatibility or sustainability [22]. Therefore, alternative materials such as poly(urethanes), poly(caprolactone) (PCL) or poly(ethylene glycol) (PEG) derivatives, which, for decades, acted as front running technomers in the medical field, are more and more being replaced by different bio-based polymers and their follow up products. This increasing interest in such polymeric products of natural origin mainly originates from their superior biocompatibility and biodegradability [7,23,24,25]. Particularly in the medical field, PHA have the potential to outperform other polymeric materials, as already assumed in earlier years [26]. However, PHA still display drawbacks in their material characteristics, such as mediocre mechanical stability, unfavorable (bio)degradation rate, or either too high or too low degree of crystallinity. Therefore, the development of advanced PHA production processes, which increasingly resorts to genetic/metabolic engineering and synthetic biology approaches [27], is steadily accompanied by the design of new composites materials, which contain PHA in combination with other compatible organic or inorganic materials. The resulting products, which display blends and composites of different composition, can improve the mechanical properties, rate of (bio)degradation, and trigger bioactivity of PHA [28,29,30,31].

Although the production of biodegradable packaging materials, e.g., for the food sector, is commonly considered the priority field for application of PHA and its follow-up products, its use in the medical, hence, the pharmaceutical, surgical, and therapeutic area, is a strongly emerging field with high potential and expected value creation [24,25,32]. Such high-value applications of PHA help to overcome their major hurdle for broad market penetration, namely cost issues. Whilst competitive costs are a factor of major importance for the commercial usage of polymers from renewable resources in large-scale-low-value applications, e.g., as bulk packaging material, advanced medical applications such as sutures, targeted tissue repair/regeneration devices, cardiovascular stents, polymer-based depots for controlled drug release or implants and others, open new doors for economically feasible implementation of thermoplastic materials, biobased and biodegradable in their nature. These niche products primarily are assessed in terms of material performance, and only in second instance in terms of production prices [4].

The subsequent sections invite the reader on a journey into biomedical applications of PHA and their follow-up products. Figure 1 provides the general chemical structure of PHA and illustrates the composition of different types of PHA discussed in this article. It has to be emphasized that this review differentiates from previous review articles dealing with medial application of PHA by being more holistic. On the one hand, the work encompasses biocompatibility aspects; on the other hand, the whole range of different reported biomedical applications of PHA is reviewed. Moreover, it summarizes diverse processing techniques, which advance the field of PHA regarding their applicability and competitiveness on the market. Therefore, the next section deals with a summary of efforts dedicated to obtain PHA at purity levels sufficient for *in vivo* use. Subsequent chapters summarize reported studies about the use of PHA as carrier material for encapsulation and controlled *in vivo* release of bioactive compounds, and, finally, the use of PHA in the surgical field and as scaffold material in tissue engineering.

## 2. Biocompatibility Aspects and Purity Requirements for PHA to be used *In Vivo*

Sufficient biocompatibility, hence, the feature of a material not to exert any negative effect on living organisms, isolated cells or their biological surroundings, is the precondition for the eligibility of an object to be implanted in the organism of humans or animals. Biocompatibility of objects used *in vivo* is determined by a range of factors, such as its chemical composition, surface porosity, shape, the target tissue where it is incorporated, and specifically its purity. As comprehensively reviewed by Zinn and colleagues, many polymeric materials traditionally used for *in vivo* applications, such as silicone, are suspected to cause malign effects like inflammation or are even suspected to be carcinogenic; this calls for new biocompatible materials, such as PHA and follow-up composites and blends [7]. It has to be emphasized that, despite the numerous *in vivo* experiments which have been carried out to date with PHA-based materials, not a single evidence for carcinogenic effects was evidenced [24].

To demonstrate the biocompatibility of PHA and its follow-up products, Hufenus et al. prepared materials obtained by melt-spinning of mixtures consisting of the PHA copolyester poly(3-hydroxybutyrate-*co*-3-hydroxyvalerate) (PHBHV) and poly(lactic acid) (PLA); obtained fibers revealed high tensile strength and were subjected to biodegradability and biocompatibility studies using human fibroblast cells. In these studies, the high cyto-compatibility of prepared PHBHV/PLA fibers was demonstrated; the cells proliferated well in parallel to progressing fiber degradation. Moreover, the increase in molecular mass of the polymers caused by the biodegradation of low molecular mass fiber domains reduced the fibers’ tensile strength by up to 33% after one month of incubation, which further evidences the high suitability of this new material for *in vivo* applications [33].

To eliminate microbial components (cell debris or metabolites) from crude PHA, the biopolymer has to be carefully purified before its processing [26]. If PHA will be biomedically used, particularly bacterial endotoxins need to be efficiently removed. Chemically, endotoxins constitute lipopolysaccharides (LPS), which are heat-resistant components produced and located in the outer cell membrane of Gram-negative microbes. Together with surface structure and shape of PHA-based items to be used *in vivo*, LPS are mainly responsible for inflammatory reactions to such biomaterials. LPS are liberated during cell lysis and product recovery and contaminate PHA, hence, LPS production severely hampers the *in vivo* applicability of PHA from Gram-negative organisms, e.g., its use for production of implants, surgical sutures, etc. (reviews by [7,25,34,35]. In contrast to Gram-negative microbes, Gram-positive bacteria do not produce lipopolysaccharides (LPS). This puts members of the genus *Bacillus* as PHA producers in a new limelight [36]. An endotoxin-free PHA was produced by the Gram-negative bacterium *Novosphingobium* sp., which was cultivated on crude glycerol as carbon source for growth and PHA biosynthesis. NMR analysis identified the PHA as PHB homopolyester. Here, cells were disrupted by sodium hypochlorite solution; the remaining PHA granules were subsequently washed with solvents of different polarity (water, ethanol and acetone); the remaining polyester was dissolved in chloroform and re-precipitated for additional purification [37].

Sevastianov et al. underlined the importance of proper endotoxin removal from such PHA specimens, which get in contact with blood. In their study, not highly purified PHA produced by the strain *Ralstonia eutropha* B5786, PHB and PHBHV copolyesters with 8 or 14 mol % 3-hydroxyvalerate (3HV) were used. In contact with human blood, films of these biopolyesters activated blood coagulation and the complement reaction, but not the hemostasis system at the level of cell response. Detailed GC-MS analysis of the substances responsible for these reactions unambiguously revealed the significant role of long chain fatty acids as typical LPS constituents. After carrying out a special purification procedure, the PHB and PHBHV samples displayed high hemocompatibility, as demonstrated by quantitative and morphological assessment of blood platelets adhesion to the surface of PHA films, by evaluation of the blood plasma recalcification time, and by complement activation studies [38].

For quantification of the LPS load of a PHA sample, a fast and expedient method was developed, which resorts to a commercially available chromogenic Limulus Amebocyte assay. This study investigated the LPS contamination of poly(3-hydroxyhexanoate-*co*-3-hydroxyoctanoate) (PHHxHO) and poly(3-hydroxy-ω-undecenoate*-co-*3-hydroxy-ω-nonenoate-*co*-3-hydroxy-ω-heptenoate) bio-synthesized by *Pseudomonas putida*. Extraction with four different solvents (methyl *tert*-butyl ether, ethyl acetate, acetone, methylene chloride) and subsequent filtration through a charcoal bed generated a colorless PHA with LPS levels below 1 endotoxin unit (EU) per gram. For PHHxHO, solubility at room temperature was 18 times higher in dichloromethane than in the halogen-free solvents, whereas extraction yields were the same for all tested solvents for the copolyester consisting of unsaturated building blocks [39].

In the context of preparing highly purified PHA, it should be noted that these biopolyesters are highly soluble in halogenated solvents such as dichloromethane, chloroform, or dichloroethane [34]. In order to avoid the use of halogenated, eco-toxic compounds, alternative solvents like ethyl acetate, butanol, pentanol, methyl *tert*-butyl ether, organic carbonates, and others [35,39,40,41], or, more recently, ionic liquids [42] have been investigated as well for PHA recovery from microbial biomass. After extraction from biomass and subsequent precipitation of PHA by solvent evaporation or precipitation in a PHA-non solvent such as hexane, ethanol, methanol, or acetone, the polyester should repeatedly be dissolved and precipitated in order to guarantee sufficient purity [34].

Post-synthetic treatment for LPS removal resorts to H_2_O_2_, ozone, sodium hypochlorite or NaOH [35,43,44], or the repeated filtration through activated charcoal; however, the resulting purities are not sufficient for *in vivo* applications. In addition, treatment with said oxidants can cause degradation of the biopolyesters [39].

Koller et al. reported the usage of acetone, a ketone traditionally described as “anti-solvent” for *scl*-PHAs, for the recovery and purification of the PHA-terpolyester poly(3-hydroxybutyrate-*co*-21.8%-3-hydroxyvalerate-*co*-5.14%-4-hydroxybutyrate) (PHB4HBHV) produced by the haloarchaeon *Hfx. mediterranei*. Simultaneous extraction and purification of the PHA terpolyester occurred in a custom made closed reactor system under high temperature and pressure above acetone’s boiling point. This extraction equipment combined components for extraction, filtration and PHA recovery, and performed at least comparable to previously reported repeated dissolution-precipitation techniques in terms of product recovery yield and purity. This new method can be applied for recovery of all types of PHA [45].

Wang et al. developed an effective extraction strategy for recovery of PHHxHO produced by the Gram-negative bacterium *Pseudomonas putida* GPo1. These authors demonstrated that 1-hexane or 2-propanol are optimal solvents to prepare PHHxHO of high purity. Using 1-hexane, PHHxHO extraction was accomplished at 50 °C, subsequent cooling to 0–5 °C resulted in polyester precipitation. Using this method, a LPS level below 15 EU per gram PHHxHO was obtained, which is substantially lower than 20 EU/g, the internationally allowed LPS limit for devices applied in medical devices [44]. For devices, which get in direct contact with cerebrospinal fluid, the limit is specified with 2.15 EU/g [32]. After re-dissolving in 2-propanol at 45 °C and subsequent cooling down to 10 °C, PHHxHO with minimal endotoxicity loads of only 2 EU/g was precipitated [46].

Another powerful tool for preparation of medical-grade PHA is supercritical fluid extraction (SFE). Williams and Martin report the high efficiency of pure supercritical CO_2_ (sCO_2_) in extracting lipophilic compounds from PHA-rich biomass. These authors proposed the use of supercritical mixtures of CO_2_ and traditional extraction solvents for extraction of extremely pure PHA at high extraction yields. Especially *mcl-*PHA shows outstanding solubility in supercritical mixtures; for example, PHHxHO extracted by supercritical solvents reached a purity level of 100% in only one single extraction step; this product contained 25–150 times less LPS than PHHxHO isolated by traditional solvent-non-solvent extraction and precipitation [32]. SFE for generation of highly pure *scl*-PHA was also investigated by Khosravi-Darani and colleagues, who studied the recovery of PHB by disruption of the Gram-negative bacterium *Ralstonia eutropha* (today: *Cupriavidus necator*) cells by sCO_2_. The impact of applied drying strategy, modifier, physiological stage of cells, repeated release of supercritical CO_2_ pressure, operating pressure, and temperature on PHB purity and molecular mass have been evaluated. PHB recovery was studied based on a combination of chemical pretreatments (NaCl or alkaline) and SFE. Cells were exposed for 1 h either to NaCl (140 mM) at 60 °C, 1 h, or to NaOH (0.2–0.8 wt. %). At least 0.4% (wt./wt.) NaOH enabled complete cell disruption, when releasing sCO_2_ pressure twice. Pretreatment with NaCl was less effective than alkali-pretreatment. Cells in growth phase were less resistant to disintegration than PHA-rich cells in the later, nutritionally limited, stage of cultivation. Moreover, products obtained from lyophilized biomass were of higher purity than PHB recovered from wet biomass. The method was proposed by the authors as economic and competitive with solvent-based recovery methods in terms of PHB recovery yield and energy consumption; moreover, the method was reported to be environmentally superior to solvent-based techniques [47].

Only recently, a study by Daly et al. demonstrated that oil residues can efficiently be removed from PHA by both sCO_2_ and “CO_2_ expanded ethanol”. Using sCO_2_, more than 70 wt. % of impurities were removed from PHB at 50 °C and high pressure (150 bar). Adding a small amount of ethanol via a “CO_2_ expanded ethanol” process, more than 93 wt. % of oil residues were removed from the biopolymer at similar temperature, but reduced pressure. The authors argue that such approach minimizes the need for organic and possibly precarious solvents, because CO_2_ and ethanol are easily recyclable. In addition, this method gets along with just a few process stages [48]. Table 1 summarizes important biocompatibility studies for different types of PHA as discussed in chapter 1.

## 3. Drug Encapsulation in PHA Carriers for Controlled Liberation of Bioactive Compounds

### 3.1. General

As reviewed by Nobes et al. the application of PHA in drug release systems was probably the first pharmaceutical-therapeutic application of these biopolyesters; this application has the longest tradition, dating back to the studies carried out by Korsatko et al. in the early 1980s [53]. These authors carried out tissue compatibility studies of parenteral PHB tablets in mice fibroblast cultures and *in vivo* in living mice [49]. The PHB used in this study was produced by *Alcaligenes eutrophus* H16 (today *C. necator* H16). In the animal experiments, it was demonstrated for the first time that PHA represents a biodegradable carrier material for delayed drug release. For the *in vitro* fibroblast cultures, PHB did not negatively affect cell growth and metabolism. Regarding the *in vivo* tests with subcutaneous PHB implants, the authors observed formation of a capsule around the PHB-implant, consisting of connective tissue, and noticed inflammatory reactions during the entire period of 20 weeks. Interestingly, this inflammatory reaction was described as positive for a rapid implant degradation and drug release [49]. Based on today’s state of knowledge, it very likely that the used PHB, although vapor- and UV-sterilized, was not of sufficient purity to prevent inflammation. Moreover, it should be noted that these studies only involved the investigation of PHB-carriers without any bioactive drug; hence, placebo pills were used. However, this study can be considered the first serious report on *in vivo* use of PHA.

### 3.2. PHA-Based Micro- and Nanocarriers

To an increasing extent, encapsulation of bioactives, such as antibiotics or therapeutic drugs resorts to the use of micro-and nanosized carriers, acting as spherical, fibrous, or rod-shaped vehicles performing the transport of bioactive compounds to target tissues. Such nanoparticles, with typical diameters in the range of 10² nm, display high overall surface area for the release of the bioactive compound, and it is possible to profit from surface interactions, which enhance the bioavailability of the drug, thus controlling the pharmacokinetic properties of the dosage form and consequently enhancing the drug’s therapeutic value. Further, enhanced tissue selectivity can be obtained by using micro- and nanoparticles for drug delivery, which originates from the selective uptake of nanoparticles in target tissues (reviewed by [54,55]).

In the context of micro-and nanocarrieres for targeted drug release, the group of Kassab and colleagues developed PHA microspheres, 120–200 µm in size, by a solvent evaporation technique, and tested them as embolization materials in dog experiments. Renal angiograms and histopathological observations demonstrated that PHB microspheres can readily be used as alternative embolization/chemoembolization agents [56]. Further, the same group of researchers studied the release of rifampicin immobilized in PHB microspheres, 5–100 µm in size. Using a gravity field-flow fractionation technique, it was possible to classify the microspheres into fractions of similar diameters. Generally, drug release occurred very fast; 90% of rifampicin was released within 24 h. However, this liberation rate strongly depended in microsphere diameter and drug loading [57].

Sendil and colleagues prepared PHBHV microspheres and microcapsules loaded with the antibiotic tetracycline both in its acidic and in neutral form. These drug release systems were produced as medication for periodontal diseases. Microcapsules of PHBHV with 3HV fractions of 7% were prepared under different conditions using water/oil/water double emulsion; their properties such as encapsulation efficiency, loading, release kinetics, and morphological properties were investigated. It was found that concentration of the emulsifiers poly(vinyl alcohol) (PVA) and gelatin (varied between 0–4%) influenced the encapsulation efficiency appreciably. Neutralizing the highly water-soluble target-antibiotic tetracycline HCl turned out to increase its encapsulation efficiency and to decrease the liberation rate. In all experiments, the authors noticed complete antibiotic release before any PHBHV degradation was observed [58].

A study performed by Xiong et al. compared the controlled intracellular drug release behavior for the lipid-soluble dye rhodamine B isothiocyanate (RBITC) encapsulated in nanoparticles of PHB, statistical PHBHHx copolyesters, and PLA. Mean nanoparticle diameters amounted to 160 nm (PHB), 250 nm (PHBHHx), and 150 nm (PLA), respectively. More than 75% of RBITC-loading was achieved with PHB and PHBHHx nanoparticles. It turned out that the nanoparticles were able to deeply penetrate into the investigated tissue material; macrophage endocytosis resulted in a sustained drug release over a period of at least 20 days for PHB and PHBHHx nanoparticles, while release from PLA nanoparticles only lasted 15 days. The type of PHA (PHB or PHBHHx, respectively) or the particle size only insignificantly effected drug-release performance [59].

Naveen et al. successfully applied an electrospinning approach to produce nanofibrous drug carriers using hexafluoroisopropanol (HFIP) as the solvent. These nanofibrous scaffolds displayed supported fast cell growth without negatively effecting cellular morphology; a cell viability of 87% was attained after 48 h. Later, these PHB nanofiber mats were loaded with the antibiotic kanamycin sulphate, which attached to the mat’s surface and inside its cavities. These antibiotic-loaded nanofibers were tested against the well-known pathogenic indicator organism *Staphylococcus aureus*; inhibition zones obtained in solid culture cultivation of the strain indicated the strong antibiotic effect of the prepared nanofibers. Within 8 h, more than 95% of the antibiotic was released [60].

In order to treat infection diseases by providing antibiotics directly at the infection site, Gursel et al. prepared PHBHV rods with varying 3HV fractions loaded with the antibiotics sulbactam: cefoperazone and gentamicin. Mimicking physiological conditions, antibiotic liberation was studied *in vitro* in phosphate saline buffer at room temperature. The release profiles were in accordance to the release patterns from monolithic specimens investigated in parallel, where a rapid initial release is followed by a slower, sustained release. Using PHBHV rods with 22 mol % 3HV, the phase of sustained release lasted for extended periods. This duration is critical because an appropriate therapy of, e.g., osteomyelitis by antibiotics requires providing the minimal effective concentration for at least 6 weeks. After *in vitro* release, voids with sharp edges occurred on the PHA rods, indicating the solvation of the antibiotic crystals; however, the polymer was not degraded within this test period. Shifting the PHA/antibiotic ratio from 2:1 to 20:1 (*w*/*w*) considerably slowed down the liberation rate. A change of the PHA composition on the level of the 3HV/3HV ratio did not result in any noticeable changes in the release profiles [61].

Similar studies were later carried out by Türesin et al. who used various statistic PHBHV copolyesters and random copolyesters of 3HB and 4HB (PHB4HB) for development of biodegradable rod-shaped implants dedicated to the local liberation of the antibiotics Sulperazone^®^ and Duocid^®^, which are used for therapeutic treatment of chronic osteomyelitis. Type of antibiotic, drug loading, and additional surface coating of the implant turned out to affect the *in vitro* drug release profile. Rate and duration of Sulperazone^®^ release from PHB4HB rods were strongly determined by the drug loading. The drug dissolution rate of the antibiotics was significantly higher than the rate of PHA degradation, which indicates that the release phenomenon was more dependent on drug dissolution rather than on PHA degradation or diffusion. When rods were coated with the same type of PHA, the initial burst effect was considerably reduced, and the release rate significantly decreased. Drug release from coated rods at a constant rate continued for more than two weeks, whereas uncoated rods released the antibiotics in less than a week. Introducing Duocid^®^ into the hydrophobic PHA matrix generated rods characterized by rather smooth surfaces; release from these rods was considerably higher than for Sulperazone^®^-loaded rods [62].

Recently, Scheithauer et al. successfully prepared PHBHV microspheres loaded with the phytoestrogen daidzein by an electrospraying technique. This new approach enabled uniform surface morphology of the microspheres and narrow particle size distribution with mean sphere diameters of about 5 µm, and did not exert shear or temperature stress to the drug; moreover, initial burst release of daidzein in the first hour was negligible, but followed by a sustained release within three days. These microspheres, with the drug daidzein being incorporated into the amorphous phase of PHBHV, were developed as an alternative osteoporosis hormone therapy [63].

Using a solvent evaporation technique, blend microspheres consisting of PHB and cellulose acetate phthalate (CAP), 29 μm to 67 μm in diameter, have been prepared by Chaturvedi et al. in mass ratios PHB/CAP = 2/1, 1/1, and 1/3 (wt./wt.). These polymers, pH-sensitive in their nature, were investigated as drug release materials for the in-colon delivery of the anticancer drug 5-fluorouracil. *In vitro* release of the encapsulated drug was studied for 2 h at 37 °C in an acidic buffer medium similar to the pH-conditions in the stomach (pH-value 1.2); subsequently, the release was monitored in a simulated intestinal medium (pH-value 7.4). It was revealed by these *in vitro* tests that the release of the drug from blend microspheres was strongly pH-dependent, which is in contrast to the release from pure PHB microspheres, indicating that this blend-based approach is more efficient for delivering 5-fluorouracil to the colon. Experimental release data were fitted to empirical equations to developed mathematical models of the drug release profile [64].

Rezaie Shirmard et al. prepared PHBHV/PVA nanospheres of 250 nm diameter via an emulsification and solvent evaporation technique, and loaded them with the therapeutic agent fingolimod. Fingolimod, a therapeutically important drug for multiple sclerosis treatment and as immunosuppressant for kidney transplantation, was encapsulated either in its highly water-soluble acidic form, or after neutralization. In the case of neutralized fingolimod, encapsulation efficiency was considerably higher. The authors report the optimum recipe to prepare nanosized particles of uniform size distribution and high encapsulation efficiency (73%) with 1.32 wt. % PHBHV, 0.42 wt. % PVA (enhances droplet formation and uniformity of particle size) and 5 mg fingolimod. Drug-release from the nanoparticles was studied over a period of one month; a characteristic triphasic release profile with an initial burst effect was monitored [54].

Masood et al. reported a similar approach based on the coating of randomly distributed PHBHV copolyester nanoparticles with different 3HV fractions by PVA. Here, it is noteworthy the PHBHV copolyesters were produced by the Gram-positive strain *Bacillus cereus* in order to generate LPS-free biopolymer. Again, these nanoparticles were prepared by an emulsification and solvent evaporation approach, and contained ellipticine, an antineoplastic drug applied in cancer therapy. Mean diameters of nanoparticles with and without drug loading were in the rage of about 200 nm, with nanoparticles containing the drug being of increased size (208–283 nm) compared to those only consisting of polymer (184–198 nm). *In vitro* cytotoxicity tests demonstrated the high biocompatibility of PHBHV nanoparticles not loaded with ellipticine; survival of a cancer cell line was not affected by the “placebo” nanoparticles. In contrast, PHBHV nanoparticles loaded with ellipticine drastically inhibited growth of cancer cells; this inhibitory effect was even higher than observed for the not encapsulated drug, which underlines the possibility to increase ellipticine’s cytotoxic effect to cancer cells by supplying it via small size particles, which increases its bioavailability [65].

Wu and colleagues reported a protocol for simple and safe preparation of random PHBHHx copolyester nanoparticles coated with sub-cytotoxic concentrations of poly(ethylene imine); this nanoparticle system, targeting different cell types, was used to study cell response in *in vitro* and *ex vivo*. Rhodamine-B-loaded PHBHHx nanoparticles of a mean size of 154 ± 71 nm were coated with poly(ethylene imine) in order to assist binding to and uptake by cells. The nanoparticles traveled along endolysosomal compartments, the endoplasmic reticulum and the Golgi complex, and did not cause any detrimental effects on cell morphology and respiration [66]. Table 2 summarizes the drug release studies using PHA-based carriers described in Section 2.

## 4. PHA-Based Implants, Sutures and Scaffolds for Tissue Engineering and Tissue Repair

### 4.1. PHA-Based Implants

Elasticity modulus, tensile strength and tensile strain of PHB and its composites are very similar to that of bone and thus promising for application as implant materials. Compared to clinically used materials such as poly(lactide-*co*-glycolid) (PLGA), poly(glycolic acid) (PGA), and PLA, PHB-based implants feature the additional benefit of an unchanged local pH value during degradation, which makes them well tolerated by immune system and cells. However, regarding the degradation rate of biodegradable implants, the high crystallinity, particularly of the most commonly used homopolyester PHB, constitutes a considerable problem, because it complicates the attack of the implants by the degrading enzymes. A study carried out by Meischel and colleagues demonstrated the rather high resistance of PHB-based implants against *in vivo* degradation in rat model experiments. Here, the response of femoral bone healing in growing rats to new PHB-based composite implants was investigated by *in vivo* micro-focus computed tomography (µCT) of the bone reaction at the implant site and the implant resorption of the implants. Implants were explanted from the femoral bones after up to 36 weeks, and scanned with high resolution *ex vivo* µCT. Moreover, the surface roughness of explanted implant pins was studied by scanning electron microscopy and energy dispersive X-ray spectroscopy in order to assess the ingrowth capability for bone tissue. Four different PHB-based composites with ZrO_2_ (used to increase radiological contrast values of the implants) and Herafill^®^ (used to increase degradation rates) were investigated. Even after 36 weeks *in vivo*, none of the implants was significantly degraded. However, the authors suggest that these materials might still be appropriate for designing custom-made 3D-printed implants, or as coatings to reduce degradability of implants dissolving too rapidly. The composite with ZrO_2_ and 30% of Herafill^®^ showed best bone accumulation behavior in vicinity of the implant. Roughness measurements and surface observation did not show any visible changes on the implant surfaces. Biomechanical characteristics such as the adhesion strength between bone and implant were obtained by measuring the shear strength and the push-out energy of the bone-implant interface. The results showed that improvement of the mechanical properties of the studied composites is still necessary to design applicable implant materials with high load-bearing capacity [67].

In the context of insufficient biodegradability of PHB-based implants, the utilization of less crystalline and better degradable co- and terpolyesters for medical applications was suggested in the past [66]. To make compromises between the excellent biocompatibility and stability of PHB and the expedient degradability but lower stability of *mcl-*PHA and diverse *scl*-PHA copolyesters, various blends of different types of PHA were investigated. Moreover, PHA was also combined with other compatible materials. As an example, Wang and associates studied the degradability of films of PHBHHx blended with different amounts of gelatin in simulated body fluid at 37 °C. These authors report that the mass loss of the blends increased with increasing gelatin contents. This was explained by the observation that blending with gelatin results in increased surface porosity and decreased crystallinity. In the blend containing 10% gelatin, the spatial structure of PHBHHx was disrupted to a lower extent, which resulted in enhanced mechanical properties, expressed as elastic modulus. Short-term mass loss of the blends was predominately caused by the loss of gelatin; however, increasing gelatin loss and the consequently increasing porosity of the test specimens are beneficial for long-term degradability of the PHBHHx. Moreover, gelatin-containing blends revealed enhanced performance on viability of mouse osteoblast cells than observed for pure PHBHHx. The authors assumed that increased surface porosity and roughness, important parameters for osteoblast attachment on biomaterials, and decreased crystallinity caused by incorporation of gelatin is favorable for cell growth if compared with neat PHBHHx, thus making it more suitable as material for medical implantation [51]. Other studies describe the utilization of composites of PHA and inorganic materials such as bioactive glass [68,69], ceramics, or hydroxyapatite for tissue engineering to improve mechanical properties, degradation rate, and to impart bioactivity; these inorganic phases can be applied either as filler material in the PHA matrix, or as coating materials (reviewed by [30]).

### 4.2. PHA in Tissue Engineering

Currently, tissue repair represents one of the major fields of medical applications for PHA. The expression “tissue engineering” described the use of biomaterials dedicated to replace damaged organs or tissue [70,71]. PHA’s high biocompatibility makes them ideal candidates for production of scaffolds, which can then be used to repair damage in various types of tissue. For example, the high biocompatibility of implants of PHB and PHBHV was successfully demonstrated in animal-model experiments. As reported by Shishatskaya et al. [50] and Volova et al. [34], the physiological and biochemical reactions of rats implanted with PHA sutures were investigated in long-term studies. One-year long monitoring showed that the animals with PHA threads were in a good health conditions and active throughout the entire experimental period; implanted polymer threads did not negatively affect the rats’ organism, as previously reported [34,70].

Ellis et al. reported a new technique for tissue repair by preparing laser-perforated biodegradable scaffold films of solvent-casted PHBHV copolyesters of statistical distribution. The dimensions of the perforations were in the µm range, which enabled human keratinocytes transferred to the films to attach and grow on the film’s surface, and, in addition, to penetrate the pores and thus to reach the damaged tissue. Mechanistically, the authors suggested a drastically decreased crystallinity at the pore edges, which contributes to the expedited cell adhesion and facilitated growth and migration of cells as desired for regenerative medicine [72].

### 4.3. PHA-Sutures for Muscle and Skin Regeneration

In order to be effective in wound closures, a polymer to be used as suture material needs to reveal excellent tensile strength [23]. By testing PHB and PHBHV sutures in animals intramuscularly, Shishatskaya et al. revealed that such sutures exhibit sufficient mechanical strength to make them suitable for treatment of muscle-fascial wounds. The tissue in contact with the sutures exhibited a transient post-traumatic inflammation reaction; further, the formation of fibrous capsules with a thickness of up to 200 μm was observed, which became thinner upon continued contact. When implanting the sutures in the animals for periods of up to one year, no suppuration or necrosis were observed, which is analogous to the benign effect of silk and catgut sutures investigated in parallel [50].

The homopolyester poly(4-hydroxybutyrate) (P4HB) displays a representative of the *scl-*PHA family quite different from its relatives like PHB or PHBHV; this polymer, consisting exclusively of achiral building blocks, displays an elongation at break up to 1000%, hence, it is extremely flexible and stretchable. For comparison, elongation at break for other biopolymers are reported with max. 3% (PGA), 3% (PHB), 6% (PLA), or 60% (PCL). As suture material, oriented P4HB fibers display higher tensile strength (545 MPa) than, e.g., poly(propylene) sutures (410–460 MPa). In addition, the Young’s modulus of P4HB sutures is significantly lower than other commercially available monofilament sutures [73]. The company Tepha Inc. (Lexington, MA, USA), commercializes several PHA-based devices for medical purposes. TephaFLEX^®^ sutures made of P4HB are the best known among these products; these sutures were successfully past approval by the US Food and Drug Administration (FDA). The *in vivo* absorption rate of P4HB amounts to 8–52 weeks, which is considerably faster than it in the case of PHB. In the Tepha process, the homopolyester P4HB is produced by a specifically engineered fermentation process using transgenic *E. coli* K12. Tepha Inc. produces additional PHA-based surgical products such as meshes and films, all of them displaying favorable mechanical properties (reviewed by [23]).

Bioactive glass displays an ideal material for hemostasis, because, upon hydration, it releases Ca^2+^ ions, which are known to support thrombosis. In a study carried out by Francis et al. bioactive glass nanoparticles were embedded in PHB microsphere films as smart materials for skin regeneration. The authors investigated the effect of the glass nanoparticles on structure, thermal properties and biocompatibility of the PHA films; moreover, by studying the hemostatic efficiency of the nanoparticles *in vitro*, the PHA films turned out to significantly reduce the time of clot detection. Studying the effect of the particle roughness caused by hydroxyapatite formation on cell adhesion, cell differentiation, and cell mobility was carried out by immersing the composite films in simulated body fluid up to one week; after some days, the surface became rough and uneven. When testing the biocompatibility of the composites with enhanced surface roughness, lower protein adsorption capacity and reduced cell adhesion were observed, indicating that surface roughness of such nanoparticles has a zenith that should not be surpassed in order to prevent deleterious effects on cell adhesion and differentiation [68]. Using a 3D-bioplotter, the team of Zhao et al. produced 3D-scaffolds of composites of PHBHHx and mesoporous bioactive glass. In *in vivo* experiments aiming at investigating these materials for enhanced bone regeneration, the robust and highly porous scaffolds featured excellent bioactivity, stimulated human bone marrow stromal cells adhesion and stimulated bone regeneration [69].

### 4.4. PHA in Blood Vessel Regeneration

Coronary artery disease is the cause of a significant number of deaths in industrialized countries. In angioplasty, a stent is usually used to extend, support and allow sufficient blood stream in narrowed blood vessels. Various restrictions of traditional metal stents, often coated with petro-plastics, could be overcome by using biodegradable alternatives. In the ideal case, biodegradable stents shall provide mechanical support and, after degradation, leaving behind merely the healed blood vessel. However, previously tested stents made of diverse biodegradable polymers revealed mechanical properties insufficient to provide the required support in the artery, can potentially damage blood vessels, and cause inflammatory reactions by generation of acidic degradation products [74]. As an outdoor, Basnett et al. developed novel blends of the highly crystalline homopolyester PHB (produced by the Gram-positive strain *Bacillus cereus* SPV) and the expediently elastomeric homopolyester poly(3-hydroxyoctanaote) (PHO) (produced by the Gram-negative strain *Pseudomonas mendocina*) as potential biological stent materials. These blends were prepared by a rather simple solvent casting method, with the ratio of PHO in the blends amounting to 20%, 50%, and 80%. If compared to neat PHO, films of these blends displayed higher tensile strength and enhanced Young’s moduli. Moreover, cell viability (tested with human microvascular endothelial cells) and protein adsorption capacity (tested with fetal bovine serum) of the blend films was significantly higher than the case of films of neat PHO homopolyester. Highest cell viability was obtained for blends with 20% PHO, the same trend was observed for protein adsorption capacity. In addition, hydrolytic degradation of blend films occurred faster than in the case of homopolyester films, and could be triggered to the optimum rate for an envisaged application in the medical field. Regarding biodegradability, it turned out that degradation of these blends takes place by surface erosion and not via bulk degradation, which enables a better controlled degradation process, while the core structure remains intact. Melting temperature and glass transition temperature by trend increased with increasing PHB content in blends. The authors describe these novel blends as highly biocompatible materials with surface roughness and thermo-mechanical stability suitable for various medical applications [75].

Recently, Puppi and colleagues developed biodegradable stents consisting of blends of microbial PHBHHx and synthetic PCL, which are intended to serve for healing small caliber blood vessels, by a new manufacturing technique. Computer-aided wet-spinning of the polymer solution, a hybrid additive manufacturing technique to process polymers dissolved in organic solvents, was used as a new approach to fabricate these novel stents. Computer-aided wet-spinning enables manufacturing scaffolds of pre-defined geometry and tailored internal architecture. During stent preparation, morphological characteristics like pore size, wall thickness, etc., were triggered by tuning the process parameters. Based on thermal analysis, it was shown that the wet-spinning process does not change the polymers’ molecular structures. PHBHHx stents revealed outstanding radial elasticity, while higher axial and radial mechanical strength was measured for PCL stents. In two weeks *in vitro* cultures, the new stents sustained proliferation of human umbilical vein endothelial cells; moreover, the stents showed exceptional thromboresistivity in contact with human blood [76]. Further *in vitro* investigation studies performed by the same group of authors revealed that PHBHHx/PCL blend scaffolds, manufactured by computer-aided wet-spinning from solutions of PHBHHx and PCL in THF, can sustain adhesion and proliferation of MC3T3-E1 murine pre-osteoblast cells [77].

### 4.5. PHA in Cartilage Repair

Puppi et al. exhaustively reviewed the use of PHA in cartilage engineering experiments [78]. In a study reported by Deng et al. rabbit articular cartilage chondrocytes were seeded on scaffolds consisting of PHB, PHBHHx, or blends of these biopolyesters, and incubated for 28 days. It was shown that the chondrocytes maintained their phenotype and proliferated on all of these scaffolds, with better results for proliferation obtained on the blends than on the neat biopolyesters [79]. As shown in a further study by Zhao et al. PHBHHx/PHB blends with a PHBHHx content of 60 wt. % revealed enhanced mechanical properties than neat PHB or PHBHHx, respectively; moreover, growth and physiological function of chondrocytes was considerably supported when using this blend [80]. As shown by Deng et al. rabbit articular chondrocytes seeded on scaffolds consisting either of PHB or PHB/PHBHHx blends revealed higher mRNA level of collagen II of chondrocytes on the blend than on the neat polymer when cultured for one week. Moreover, PHBHHx/PHB blend scaffolds were better suitable to anchor type II collagen filaments and to allow the filaments penetrating into internal layers. Later, the performance of lyophilized PHBHHx scaffolds in rabbit articular cartilage defect model was investigated. Here, engineered cartilage specimens were designed by seeding articular chondrocytes into PHBHHx scaffolds, and implanted for four months. These implants were shown to successfully fill the cartilage defects by forming a white cartilaginous tissue, which displayed excellent subchondral bone connection. When compared with bare PHBHHx scaffolds, the scaffold carrying chondrocytes revealed enhanced surrounding cartilage infusion, improved surface integrality, and higher accumulation of extracellular matrix components [81]. A further study compared the attachment of rabbit chondrocytes in PHBHV films obtained on the one hand by solvent casting, and, on the other hand, by electrospinning, which generated nanofibrous PHBHV mats. This study, performed by Lee et al. showed that chondrocytes attach better on the surface of electrospun nanofibrous meshes, and display a more diversified morphology [82]. In a very recent study by Mota et al. PHBHHx scaffolds were produced by so-called “additive manufacturing” using a computer-controlled wet-spinning system. Based on a layer-by-layer approach, this additive manufacturing technique enabled the fabrication of three-dimensional scaffolds with controllable fiber alignment and fully interconnected porous networks; porosity of the scaffolds was in the range of 79–88%, with the diameter of fibers amounting to 47–76 µm, and the pore size ranging from 123–789 µm. As a result of the phase-inversion process during solidification of the PHBHHx solution, the obtained fibers showed an internal porosity structure well connected to the external fiber surface. It was shown that the scaffold compressive modulus and the yield stress and yield strain can be adjusted to a certain extend by varying the architectural parameters. MC3T3-E1 murine pre-osteoblast cells were used for cell cultivation experiments on the additively manufactured PHBHHx scaffolds; after three weeks of cultivation, they displayed respectable proliferation and differentiation towards an osteoblast phenotype [83]. As a follow up, these authors used the computer-aided wet spinning method for fabrication of PHBHHx tissue engineering scaffolds with anatomical shape and customized porous structure for bone regeneration studies. Morphological and thermomechanical characterization was performed to assess the impact of the manufacturing process on material properties, and to compare the potential of PHBHHx scaffolds with anatomical star PCL scaffolds. It turned out that the scaffolds, 3D-interconnected networks of pores, were composed by overlapping microfiber layers with a sponge-like morphology. The molecular structure of processed PHBHHx was not affected by the employed technique. By studying the compressive and tensile mechanical properties of the PHBHHx scaffolds, it turned out that the porous structure revealed anisotropic behavior, and that formed macro-channels enhance the scaffold’s compressive stiffness. Moreover, PHBHHx scaffolds outperformed PCL scaffolds in terms of higher compressive stiffness and enhanced tensile deformability [84].

### 4.6. PHA in Nerve Repair

Currently, the study of nerve repair mechanisms is a hot topic in the field of regenerative medicine. Peripheral nerve injuries, e.g., caused by spinal damage, are frequently occurring and often result in permanent disability of affected people. Application of nerve conduits consisting of diverse (bio)materials for nerve repair is increasingly considered an alternative to the traditional use of autologous nerve grafts, which suffer from limited availability of donor tissues and often cause local pain at the donor operative site [85]. Already in 2002, PHB was proposed by Young et al. as a bioresorbable conduit material for the production of guidance channels to be used for long-gap bridging in peripheral nerves; in rabbit model experiments, these authors demonstrated the positive effect of PHB for long-gap nerve injury repair [86]. Later, Mohanna et al. compared PHB conduits to bridge two to four cm nerve gaps in peroneal nerves of rabbits; the PHB conduits were either empty, or contained the glial growth factor or an alginate matrix. It was shown that PHB containing the growth factor significantly increased nerve regeneration after 63 days of trial [87]. Apart from the homopolyester PHB, the positive impact of PHA copolyesters for peripheral nerve tissue engineering was demonstrated by Bian et al. who investigated porous nerve conduits consisting of PHA copolyester of 3HB and 3HHx in rat experiments; studying the impact of the implants on the animals and the implants morphology after up to three months *in vivo*, the authors underlined the expedient mechanical properties, high nerve regeneration ability, and non-toxicity of the copoyester’s degradation products [85]. Later, Wang and colleagues compared a PHA copolyester consisting of 3HB and 3HHx, a PHA terpolyester consisting of 3HB, 3HV and 3HHx, and PLA for their practical use in differentiation of human bone marrow mesenchymal stem cell (hBMSC) into nerve cells. In this study, 2D-scaffolds of the three biopolyesters, and, in addition, a 3D-scaffold of the terpolyester were produced and compared. The terpolyester films displayed better cell adhesion, proliferation and differentiation for the stem cells compared with PLA and the PHBHHx copolyester. Moreover, 3D-scaffolds better promoted the differentiation of hBMSC into nerve cells than observed for 2D-membrane films of the same material. Although smaller pore sizes of scaffolds increased differentiation of hBMSC into nerve cells, decreased cell proliferation was observed. The authors suggested the use of PHBHVHHx scaffolds with pore sizes of 30–60 μm for nerve tissue engineering to cure nerve injuries [46].

Because the regeneration effect of nerve guidance conduits based on PHBHHx and PHBHVHHx is not statistically comparable with the effect obtained by autologous nerve grafting, Lizarraga-Valderrama et al. used blends of PHB and PHO [52], as reported previously by Basnett et al. to study their suitability for nerve repair [75]. The blends used in this study contained PHO contents of 25%, 50% and 75%. They were compared with the performance of neat PHB and PHO regarding their chemical, material and biological properties in order to assess their potential applicability as base materials for nerve tissue engineering. As shown by DSC analysis, the two homopolyesters formed immiscible blends. All of the blends were biocompatible with NG108-15 neuronal cells, with the blend containing 25% PHO showing significantly better support for cell growth and differentiation, and mechanical properties suitable to use it as base material for production of nerve guidance conduits [52]. Table 3 summarizes the different types of PHA discussed in Section 4 of this review.

## 5. Conclusions

The article familiarizes readers with the definitely rich real potential biomedical applications of PHA and enhanced follow-up products. The synergism between the high biocompatibility of these microbial materials, the extraordinary adjustability of their stiffness, strength, elasticity, crystallinity, degradability, etc., *in statu nascendi* by adapting the bioprocess (strain and feedstock section), and the emerging knowledge of novel techniques to process PHA into custom-made devices makes it very likely that these multi-facetted biopolyesters can become widely accepted and implemented workhorses to treat diverse diseases and injuries in a not too distant future. However, a lot of R&D work still needs to be dedicated to design and evaluate to optimum PHA-based formulation for defined applications in this steadily emerging field. At first, additional efforts need to be dedicated to make PHA production economically more attractive; this includes the selection of inexpensive fermentation substrates, and the optimization of the cultivation regimes in order to enhance productivity; the latter refers to the large-scale implementation of continuously operated production facilities, which enable both high-throughput production of PHA-rich biomass and the subsequent downstream processing without unproductive time currently need for preparation and post-treatment of the bioreactors. Smart operation of continuously operated PHA production facilities might also pave the way to trigger the intramolecular structure of the biopolyesters, which might allow the production of biomaterials with novel, predefined properties; in this context, production of PHA in continuously operated multistage bioreactor cascades enables the synthesis of blocky structured PHA (*b*-PHA) with alternating soft and hard segments [88]; not available yet on a significant scale, this new class of biopolyesters might have intriguing material features, also benefical for the use in the biomedical field. Moreover, additional “green solvents” are awaiting their exploration for the efficient preparation of highly pure PHA. 

## Figures and Tables

**Table 1:** Different types of highly purified PHA described in this review used for biocompatibility studies.

Type of PHA	Application	Ref.
Poly(3-hydroxybutyrate) (**PHB**) (Homopolyester; *scl*-PHA)	Tissue compatibility studies of parenteral PHB tablets in mice fibroblast (*nota bene:* PHB was presumably not of high purity)	[49]
	Study of physiological and biochemical reactions of rats implanted with PHB sutures	[34]
	Preparation of highly pure PHB	[37,47,48]
Poly(3-hydroxybutyrate-*co*-3-hydroxyvalerate) (**PHBHV**) (Copolyester; *scl*-PHA)	Biocompatibility tests of PHBHV/PLA fibers	[33]
	Blood coagulation, complement reaction, and hemostasis tests	[36]
	Study of physiological and biochemical reactions of rats implanted with PHBHV sutures	[34,50]
Poly(3-hydroxybutyrate-*co*-3-hydroxyvalerate-*co*-4-hydroxybutyrate) (**PHB4HBHV**) (Terpolyester; *scl*-PHA)	Preparation of highly pure PHB4HBHV	[45]
Poly(3-hydroxybutyrate-*co*-3-hydroxyhexanoate) (**PHBHHx**) (Copolyester; *scl*-*mcl*-PHA)	Viability of mouse osteoblast cells on PHBHHx films and films of PHBHHx and gelatin	[51]
Poly(3-hydroxyoctanoate) (**PHO**) (Homopolyester; *scl*-PHA)	Biocompatibility studies with NG108-15 neuronal cells for nerve tissue engineering	[52]
Poly(3-hydroxyhexanoate-*co*-3-hydroxyoctanoate) (**PHHxHO**) (Copolyester; *mcl*-PHA)	Preparation of highly pure PHHxHO with low endotoxin levels	[39,46]
Poly(3-hydroxy-ω-undecenoate*-co-*3-hydroxy-ω-nonenoate-*co*-3-hydroxy-ω-heptenoate) (Copolyester; *mcl*-PHA)	Preparation of highly pure unsaturated PHA with low endotoxin levels	[39]

**Table 2:** Different types of PHA described in this review used for drug release studies.

Type of PHA	Application	Ref.
Poly(3-hydroxybutyrate) (**PHB**)(Homopolyester; *scl*-PHA)	Release of rifampicin immobilized in PHA microspheres	[57]
	Sustained rhodamine B isothiocyanate release by macrophage endocytosis	[59]
	Nanofibrous scaffolds for kanamycin release to prevent infection by *Staphylococcus aureus*	[60]
	In-colon delivery of the anticancer drug 5-fluorouracil from PHB/cellulose acetate phthalate microspheres prepared by solvent casting	[64]
Poly(3-hydroxybutyrate-*co*-3-hydroxyvalerate) (**PHBHV**)(Copolyester; *scl*-PHA)	Release of tetracycline immobilized in PHBHV microspheres and microcapsules	[58]
	PHBHV rods loaded with sulbactam:cefoperazone and gentamicin for sustained antibiotic release	[61]
	PHBHV/PVA nanospheres for in-colon delivery of the anticancer drug 5-fluorouracil	[64]
	PHBHV/PVA nanospheres loaded with fingolimod to treat multiple sclerosis	[54]
	PHBHV nanospheres coated with PVA for release of antineoplastic drug ellipticine (cancer therapy)	[65]
Poly(3-hydroxybutyrate-*co*-4-hydroxybutyrate) (**PHB4HB**)(Copolyester; *scl*-PHA)	Local release of antibiotics Sulperazone^®^ and Duocid^®^ for treatment of chronic osteomyelitis	[62]
	Microspheres loaded with the phytoestrogen daidzein prepared by electrospraying for osteoporosis hormone therapy	[63]
Poly(3-hydroxybutyrate-*co*-3-hydroxyhexanoate) (**PHBHHx**)(Copolyester; *scl*-*mcl*-PHA)	Sustained rhodamine B isothiocyanate release by macrophage endocytosis	[59]
	Rhodamine-B-loaded PHBHHx nanoparticles coated with poly(ethylene imine) to study *ex vivo* and *in vivo* cell response	[66]
Poly(3-hydroxyoctanoate) (**PHO**)(Homopolyester; *scl*-PHA)	Biocompatibility studies with NG108-15 neuronal cells for nerve tissue engineering	[52]

**Table 3:** Different types of PHA described in this review used for application as implants, for tissue engineering, as sutures, and for blood vessel, cartilage and nerve repair.

Type of PHA	Application	Ref.
Poly(3-hydroxybutyrate) (**PHB**) (Homopolyester; *scl*-PHA)	Bioactive glass nanoparticles embedded in PHB microsphere films for skin regeneration	[68]
	Guidance conduit channels for long-gap bridging in peripheral nerves in rabbit model	[86,87]
	Investigating biomechanical properties, osteoinduction, and *in vivo* degradability of PHB-ZrO_2_-Herafill^®^ implants in rat model	[67]
	Blends of PHB and PHO for preparation of blood vessel stents	[75]
Poly(3-hydroxybutyrate-*co*-3-hydroxyhexanoate) (**PHBHHx**) (Copolyester; *scl*-*mcl*-PHA)	PHBHHx/PCL blends prepared by computer-aided wet-spinning for production of small caliber blood vessel stents	[76]
	PHBHHx/PHB blends as scaffolds for chondrocytes proliferation	[78,79,80,81]
	PHBHHx scaffolds prepared by computer-aided wet-spinning for pre-osteoblast proliferation to osteoblasts	[77]
	Conduits for peripheral nerve tissue engineering in rat model experiment	[85]
	Scaffolds for differentiation of human bone marrow mesenchymal stem cells	[46]
	3D-scaffolds of composites of PHBHHx and mesoporous bioactive glass for bone regeneration	[69]
Poly(3-hydroxybutyrate-*co*-3-hydroxyvalerate-*co*-3-hydroxyhexanoate) (**PHBHVHHx**) (Terpolyester; *scl*-*mcl*-PHA)	Scaffolds for differentiation of human bone marrow mesenchymal stem cells	[46]
Poly(4-hydroxybutyrate) (**P4HB**) (Homopolyester; *scl*-PHA)	Highly tensile and strong suture material (TephaFLEX^®^)	[23]
Poly(3-hydroxyoctanoate) (**PHO**) (Homopolyester; *scl*-PHA)	Blends of PHB and PHO for preparation of blood vessel stents	[75]
